# A low-carbon high inulin diet improves intestinal mucosal barrier function and immunity against infectious diseases in goats

**DOI:** 10.3389/fvets.2022.1098651

**Published:** 2023-01-11

**Authors:** Chunmei Yuan, Shuiping Wang, Kefyalew Gebeyew, Xin Yang, Shaoxun Tang, Chuanshe Zhou, Nazir Ahmad Khan, Zhiliang Tan, Yong Liu

**Affiliations:** ^1^Key Laboratory for Agro-Ecological Processes in Subtropical Region, National Engineering Laboratory for Pollution Control and Waste Utilization in Livestock and Poultry Production, Hunan Provincial Key Laboratory of Animal Nutritional Physiology and Metabolic Process, Institute of Subtropical Agriculture, The Chinese Academy of Sciences, Changsha, Hunan, China; ^2^University of the Chinese Academy of Sciences, Beijing, China; ^3^Chongqing Key Laboratory of Herbivore Science, College of Animal Science and Technology, Southwest University, Chongqing, China; ^4^Department of Animal Nutrition, The University of Agriculture, Peshawar, KP, Pakistan

**Keywords:** inulin, intestinal immunity, growth performance, *mTOR* pathway, weaned goats

## Abstract

**Introduction:**

Abrupt weaning is a major stressful event, contributing to intestinal abnormalities and immune system dysfunction in weaned kids. Inulin is a prebiotic fiber with many positive functions, including promoting intestinal fermentation and enhancing host immunity in monogastric animals. However, the effects of a high-inulin, energy-rich diet on ruminal fermentation characteristics, methane emission, growth performance, and immune systems of weaned kids have not been investigated.

**Methods:**

A fully automated *in vitro* fermentation system was used to investigate ruminal fermentation characteristics and methane emission of a mixed substrate of inulin and fat powder (1.31: 1) in comparison with maize grain-based starter concentrate. During a 1-week adaptation and 4-week trial phase, 18 weaned kids (8.97 ± 0.19 kg) were randomly assigned to two groups, one with a conventional diet (83% maize grain; CON) and the other with a low-carbon, high-inulin diet (41.5% maize grain, 14.4% fat powder, 18.9% inulin; INU).

**Results:**

In the *in vitro* rumen fermentation experiment, the total gas production was not different (*p* > 0.05); however, a lower (*p* < 0.05) methane production was observed for INU as compared to CON. The average daily gain and the ratio of feed intake and growth performance of kids fed with INU were higher (*p* < 0.05) than those fed with CON. Serum concentrations of alanine transaminase (ALT) and lactate dehydrogenase (LDH) were lower (*p* < 0.05), whereas the concentration of high-density lipoprotein (HDL) and cholesterol (CHOL) were higher (*p* < 0.05) in kids fed with the INU diet as compared CON. Dietary inulin significantly increased (*p* < 0.05) the secretion of immunoglobulins (IgA, IgG, and IgM) and inflammatory cytokines (IFN-γ and IL-10) in ileum tissue. Although no differences (*p* > 0.05) were observed in mRNA expression of tight junction markers, the INU diet tended to increase (*p* = 0.09) gene expression of ribosomal protein S6 kinase beta-1 (*P70S6K*) in the mammalian target of rapamycin (*mTOR*) pathway of *longissimus dorsi* muscle.

**Conclusion:**

Our findings highlighted that a low-carbon high-inulin energy-rich diet could be used as a promising strategy to improve gut immunity and growth performance of weaned kids under abrupt weaning stress and reduce methane production.

## 1. Introduction

The growing shortage and rising prices of starch-rich feed resources have triggered an enormous interest in exploration of unconventional alternate feed resources for livestock feeding. Inulin is a naturally stored carbohydrate in the roots and rhizomes of more than 36,000 plants that act as an energy reservoir (1). However, compared to the energy-rich, 6-carbon glucose polymer of starch, inulin is a polymer of 5-carbon fructose monomers (fructans), with an extremely low energy content (4 vs. 1.5 calories/g). Inulin, as a prebiotic, has been shown to promote beneficial microbiota and improve digestive health, support weight loss, and control diabetes in humans (2). Unlike starch, inulin cannot be digested in the upper part of the gastrointestinal tract of monogastric animals, however, it is extensively hydrolyzed and fermented by the colon and rumen microbiome. Inclusion of inulin in high or low- concentrate diets of finishing beef cattle, improved rumen microbiota and the rate of feed fermentation (3). Inulin supplementation (200 g/d) to dairy cows improved rumen fermentation and relative abundance of SCFA-producing bacteria, as well as increased milk yields and milk protein and lactose content (4). Moreover, a recent study reported that increasing the inclusion levels of inulin up to 350 g/day per cow in mid-lactation Holstein dairy cows, linearly increased acetate, propionate and butyrate concentrations in the rumen fluid, milk yield, and milk fat concentration (5). In calves, however, inulin intake (18.7 g/day per calf) did not improve growth performance, which may be related to the lower energy density and weight loss property of inulin based diet (6). It is now well established from several studies that inulin can improve digestive health and production performance of adult ruminants, and the response is dose dependent. Whereas, in young ruminants, the positive effects of inulin feeding are not fully known, particularly with additional supply of energy, which warrants further systematic investigations.

Abrupt weaning is one of the most stressful events in a goat's life, because of the social separation from the mother and a sudden transition to a completely different environment and feeding regimes. The process of abrupt weaning can contribute to intestinal and overall immune system dysfunctions, leading to a decline in health, feed intake, growth performance, and increased morbidity and mortality in kids (7, 8). Several nutritional strategies have been identified to minimize the stress associated with abrupt weaning, such as optimizing energy levels of the diet and using feed additives (probiotics, prebiotics, SCFA, phytogenic compounds and nucleotides) post-weaning (7, 9). Several studies (10–14) have reported the beneficial effects of several prebiotics such as inulin, fructans, fructo-/galacto-oligosaccharides, and resistant starch on weaning animals, such as boosting intestinal immunity, regulating microbial fermentation, and modulating intestinal tight junction in weaned piglets. In recent years, there has been a growing body of literature that highlights the importance of prebiotics for easing the impact of weaning stress in young animals.

Despite the importance of inulin in preventing abrupt weaning stress, there remains a paucity of evidence on the effects of inulin inclusion in the diet on weaning stress in kids. Using a high-inulin diet supplemented with fat powder, we constructed a high-inulin energy-rich diet for comparison with conventional maize grain-based starter concentrates. This diet provided additional prebiotic protective functions along with energy for weaned kids. The purpose of our study was to investigate whether a low-carbon high-inulin, energy-rich diet could have a beneficial effect on growth performance, intestinal immunity, and mucosal barrier function in weaned kids, providing new insights for addressing the stress in weaned kids.

## 2. Materials and methods

All the protocols for the use of animal and experimental procedures in this study were approved (No. CAS-ISA-2019-0620) by the Animal Care Committee, according to the Animal Care and the Use Guidelines of the Institute of Subtropical Agriculture, Chinese Academy of Sciences, Changsha, China.

### 2.1. *In vitro* rumen fermentation

Two adult Xiangdong black does (16.77 ± 1.23 kg) with permanent rumen fistula were used as donors for the collection of rumen fluid. Each animal was fed with 300 g of rice straw and 200 g of concentrate (maize 47.0%, soybean meal 24.0%, wheat bran 22.0%, salt 0.77%, calcium carbonate 2.23%, and premix 4.00%). The goats received the feeds twice daily (8:00 and 17:00 h) and had free access to drinking water. Before morning feeding, rumen fluid was collected, filtered and mixed with a buffer solution as described by Wang et al. (15).

The *in vitro* fermentation was performed using two types of substrates, a conventional maize grain-based concentrate (CON substrate) and an inulin and fat powder blend (1.31: 1, i.e., 0.2838 g inulin and 0.2162 g fat powder in 0.5 g substrate) based substrate (INU substrate). Four replicate subsamples (0.50 g) of each substrate were incubated in fermentation bottles in two runs. The buffered-ruminal culture solution was mixed, and 60 mL was dispensed into each fermentation bottle and flushed with CO_2_ to ensure anaerobic condition. The fermentation bottles were placed in the incubator of an automated fermentation system (16) for 36 h fermentation. Data on total gas, methane and hydrogen gas production were recorded according to the procedure of Wang et al. (15).

### 2.2. *In vivo* experimental design and animal management

Eighteen weaned kids with similar initial body weight (BW; 8.97 ± 0.19 kg) were selected for the experimental trial, and randomly assigned to a maize grain (83%) based (control; CON), and inulin (18.9%) and fat (14.4%) mixture based (INU) concentrate. The ingredients and nutrient composition of two concentrates are shown in Table 1. Alfalfa hay (crude protein (CP), 14.5%; neutral detergent fiber (NDF), 37.2%; acid detergent fiber (ADF), 31.6%; ash, 6.47%; ether extract (EE), 2.63% on dry matter (DM) basis) was used as a forage source for both groups. The concentrate and forage were offered separately in a ratio of 6:4. The experiment lasted for 5 weeks, including a 1-week adaptation and 4-weeks experimental phase. The kids were raised in individual pens, fed with 180 g concentrate twice daily (9:00 and 17:00), and had free access to clean drinking water.

**Table 1:** The ingredients and nutrient composition of experimental concentrates fed to weaned kids.

**Ingredients (%)**	**CON**	**INU**	**Nutrients^b^ (DM, %)^c^**	**CON**	**INU**
Maize grains	83.0	41.5	ME (MCal/kg)	3.242	3.525
Inulin	0	18.9	CP	12.501	12.499
Fat powder	0	14.4	Na	0.814	0.806
Soybean meal	12.0	20.2	Ca	0.674	0.693
CaHPO_4_• 2H_2_O	1.5	1.5	P	0.569	0.507
CaCO_3_	0.7	0.7	Starch	60.756	30.378
NaCl	0.8	0.8	Polyfructans (inulin)	0	18.900
Premix^a^	2.0	2.0			
Total	100.0	100.0			

a The premix provided the following per Kg of concentrates: 15.33 g MnSO_4_·H_2_O, 30 g FeSO_4_·7H_2_O, 25.33 g CuSO_4_·5H_2_O, 15.33 g ZnSO_4_·H_2_O, 6.67 mg I2, 6.67 mg Se, 6.67 IU Co, 80 IU Vitamin E, 10 mg Vitamin K3, 10 mg Vitamin B1, 25 mg Vitamin B2, 8 mg Vitamin B6, 0.075 mg Vitamin B12, 0.60 mg biotin, 5 mg folic acid, 100 mg nicotinamide, and 50 mg pantothenic acid.

b ME, metabolizable energy; DM, dry matter; CP, crude protein; CON, control concentrate, INU, inulin concentrate.

c % of DM unless otherwise stated.

### 2.3. Samples collection

On the last morning of the experiment, blood sample (10 mL) was collected from the jugular vein of each kid, and centrifuged at 4°C and 1,200 g for 10 min to obtain the serum. The serum was then immediately stored at −80 °C until further analysis. At the end of experiment, all kids were euthanized by intravenous administration of pentobarbital sodium (50 mg/kg BW), and tissues samples from distal ileum and *longissimus dorsi* muscle were collected immediately. A piece of distal ileum was washed with pre-cold saline (0.9% NaCl), frozen in liquid nitrogen, and then stored at −80°C for further analysis, while the other piece was immediately placed in 4% paraformaldehyde for histomorphological analysis.

### 2.4. Sample analysis

The concentrations of total protein (TP, Roche #03183734190), serum albumin (ALB, Roche #03183688122), aspartate aminotransferase (AST, Roche #20764949322), γ-glutamyl transferase (GGT, Roche #03002721122), lactate dehydrogenase (LDH, Roche #03004732122), glucose (GLU, Roche #04404483190), triglyceride (TG, Roche #20767107322), cholesterol (CHOL, Roche #04399803190), high-density lipoprotein (HDL, Roche #04399803190), and low-density lipoprotein (LDL, Roche #03038866322) in serum were determined by ROCHE Cobas C311 automatic biochemical instrument (Roche, Switzerland). Immunoglobulins, including immunoglobulin A (IgA, FANKEW F2869-A), immunoglobulin G (IgG, FANKEW F3889-B), immunoglobulin M (IgM, FANKEW F3868-B), and inflammatory factors, including interleukin 10 (IL-10, FANKEW F3851-B), interleukin 6 (IL-6, FANKEW F3885-B), interferon-gamma (INF-γ, FANKEW F3848-B), and tumor necrosis factor-alpha (TNF-α, FANKEW F3849-B), in the ileal tissue were determined using ELISA kits (FANKEW ELISA Kit, Nanjing, China) by the standard enzyme analyzer (Rayflu-6100) according to the manufacturer's instruction.

### 2.5. RT-qPCR

RNA was extracted from ileal tissues and *longissimus dorsi* muscle using a TaKaRa MiniBEST Universal RNA Extraction Kit (Cat. #9767) following the manufacturer's protocol. RNA concentrations were measured by Thermo NanoDrop (Thermo ND-2000, MA, United States). The purification and complementary DNA (cDNA) synthesis of RNA were performed with PrimeScript RT reagent Kit with gDNA Eraser (TaKaRa Cat. #RR047A). Gene expression analysis was performed by qPCR using Roche LightCycler 480II on the LightCycler 480 (version 1.5) Real-Time PCR System. The 2^ΔΔCt^ method was used to calculate the relative gene expression of the target gene in the INU group compared to the Ct value of the housekeeping gene (β*-actin*) and the average Ct value of the corresponding target gene in CON group (17).

We designed primers based on Primer 5.0 to study the expression of genes in the *mTOR* signaling pathway of *longissimus dorsi* muscles (*PI3K, AKT, mTOR, P70S6K, 4EBP*-1, and FASN) and genes associated with ileal tight junction barrier properties (*claudin, occludin*, and *ZO*-1). All primer sequences are listed in Table 2. Primers were commercially synthesized by Sangon Biotech (Shanghai) Co, Ltd.

**Table 2:** Primer sequences of target genes used for RT-qPCR.

**Target genes**	**Primer sequences**	**PCR products (bp)**	**Reference/Gene bank accession**	**Annealing temperature(°C)**	**Amplification efficiency (%)**
*Claudin-*1	5′-CACCCTTGGCATGAAGTGTA-3′ 3′-AGCCAATGAAGAGAGCCTGA-5′	216	XM_005675123.3	63.1	97.91
*Occludin*	5′-GTTCGACCAATGCTCTCTCAG-3′ 3′-CAGCTCCCATTAAGGTTCCA-5′	200	XM_018065677.1	62.7	98.83
*ZO-*1	5′-CGACCAGATCCTCAGGGTAA-3′ 3′-AATCACCCACATCGGATTCT-5′	163	XM_018066114.1	62.2	97.08
*FASN*	5′-GTGTGGTACAGCCCCTCAAG-3′ 3′-ACGCACCTGAATGACCACTT-5′	110	XM_015098375.1	65.2	96.87
*mTOR*	5′-TGGTTTGGTGAAACCAGAGG-3′ 3′-AGAGCTGAACTTTCTGACCAT-5′	294	NM_001285748.1	62.3	96.19
*AKT*	5′-AGGCCAAGTCCTTGCTCTC-3′ 3′-ATCATCTGGGCTGTGAACTC-5′	217	NM_001382431.1	62.7	99.62
*4EBP*1	5′-GGAACTCACCTGTGACCAAA-3′ 3′-ATGGCTGGTGCTTTAAATGTC-5′	182	NM_004095.4	62.3	99.72
*P70S6K*	5′-CTCTCAGTGAAAGTGCCAAC-3′ 3′-TTGGAAGCGGTGCTGAA-5′	297	NM_001285641.1	61.7	97.93
*β-actin*	5′-GTGACGTTGACATCCGTAAAGA-3′ 3′-GCCGGACTCATCGTACTCC-5′	245	XM_018039831.1	63.5	99.55
*PI3K*	5′-TCCAGCACATGAACGTAAACAG-3′ 3′-GAAACTCGGTGACTCACACAC-5′	143	XM_028674370.1	63.9	96.65

### 2.6. Statistical analysis

The tests for significance of sources of variation and inter-rater reliability were analyzed using R language (https://www.r-project.org) (18) with tidyverse and dplyr, while figures were drawn with ggplot2 package. Results were presented as means and standard errors for tables. The significance level was set at *p* < 0.05.

## 3. Results

### 3.1. *In vitro* rumen gas production

**Figure 1:**
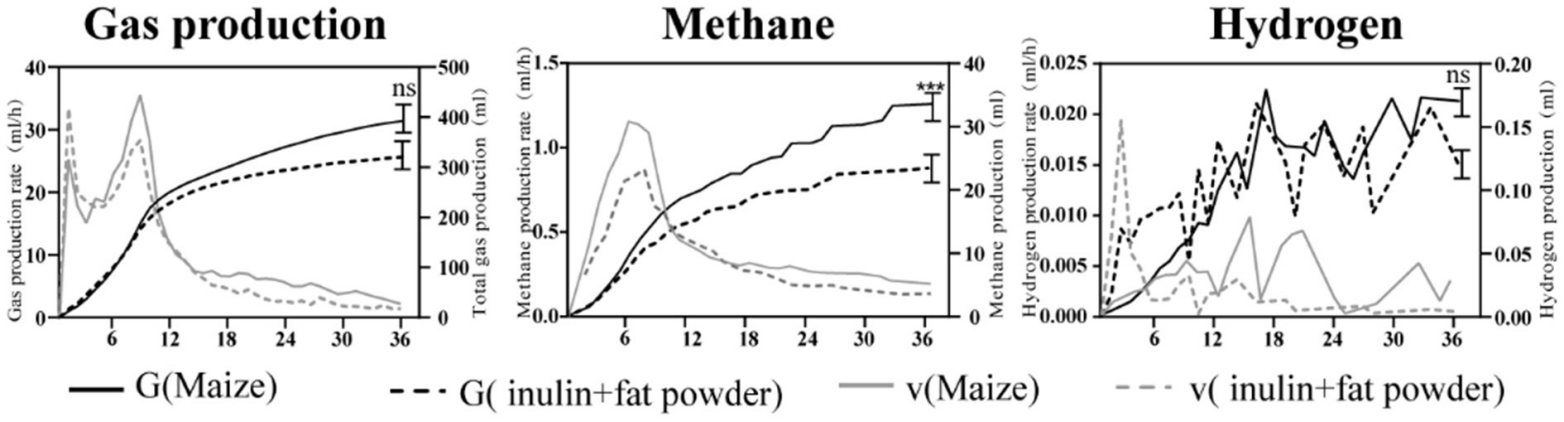
Gas production and gas generation rate of *in vitro* rumen fermentation. G(Maize), gas production by 0.5 g maize-grain based substrate; v(Maize), generation rate of gas production of 0.5 g maize-grain based substrate; G(inulin+fat powder), gas production of 0.5 g inulin-fat mixture (0.2838 g inulin, 0.2162 g fat powder) based substrate; v(inulin+fat powder), gas generation rate of 0.5 g inulin-fat mixture (0.2838 g inulin, 0.2162 g fat powder) based substrate. ***Indicates that *p*-value is < 0.001.

A summary of the *in vitro* fermentation results is shown in Figure 1. In comparison with maize-grain based concentrate (CON), the mixture of inulin and fat powder (INU) showed no differences (*p* > 0.05) in total gas and hydrogen production, but remarkably reduced (*p* < 0.001) methane production.

### 3.2. Growth performance

Compared to CON, feeding of the low-carbon, high inulin (INU) diet to weaned kids increased (*p* < 0.05) the average daily gain (ADG) by 43.27% and significantly reduce the F/G (Table 3). Moreover, the average daily feed intake (ADFI) tended to be higher (*p* < 0.07) in the INU group as compared to the CON. However, there were no significant differences (*p* > 0.05) in final weight (FW) between the diets.

**Table 3:** The growth performance of weaned kids fed low-carbon high-inulin (INU) and maize-grain based control (CON) diets, respectively.

**Items^a^**	**Groups**		**SEM**	***p*-value^b^**
	**CON**	**INU**		
IW, kg	9.19 ± 0.53	8.84 ± 0.33	0.31	0.584
FW, kg	10.77 ± 0.78	12.01 ± 0.53	0.48	0.207
ADFI, g/d	259.21 ± 26.57	321.35 ± 6.23	16.79	0.064
ADG, g/d	86.83 ± 10.27^b^	124.40 ± 11.23^a^	8.78	0.027
F/G	6.34 ± 0.62^a^	4.52 ± 0.22^b^	0.38	0.011

a IW initial body weight; FW, final body weight; ADFI, average daily feed intake; and ADG, average daily gain; F/G represents the ratio of feed intake and growth performance.

b Mean values without or with the same superscript letter in the same row mean no significant difference (*p* > 0.05), while the different superscript letters represent a significant difference (*p* < 0.05).

### 3.3. Serum metabolites

**Figure 2:**
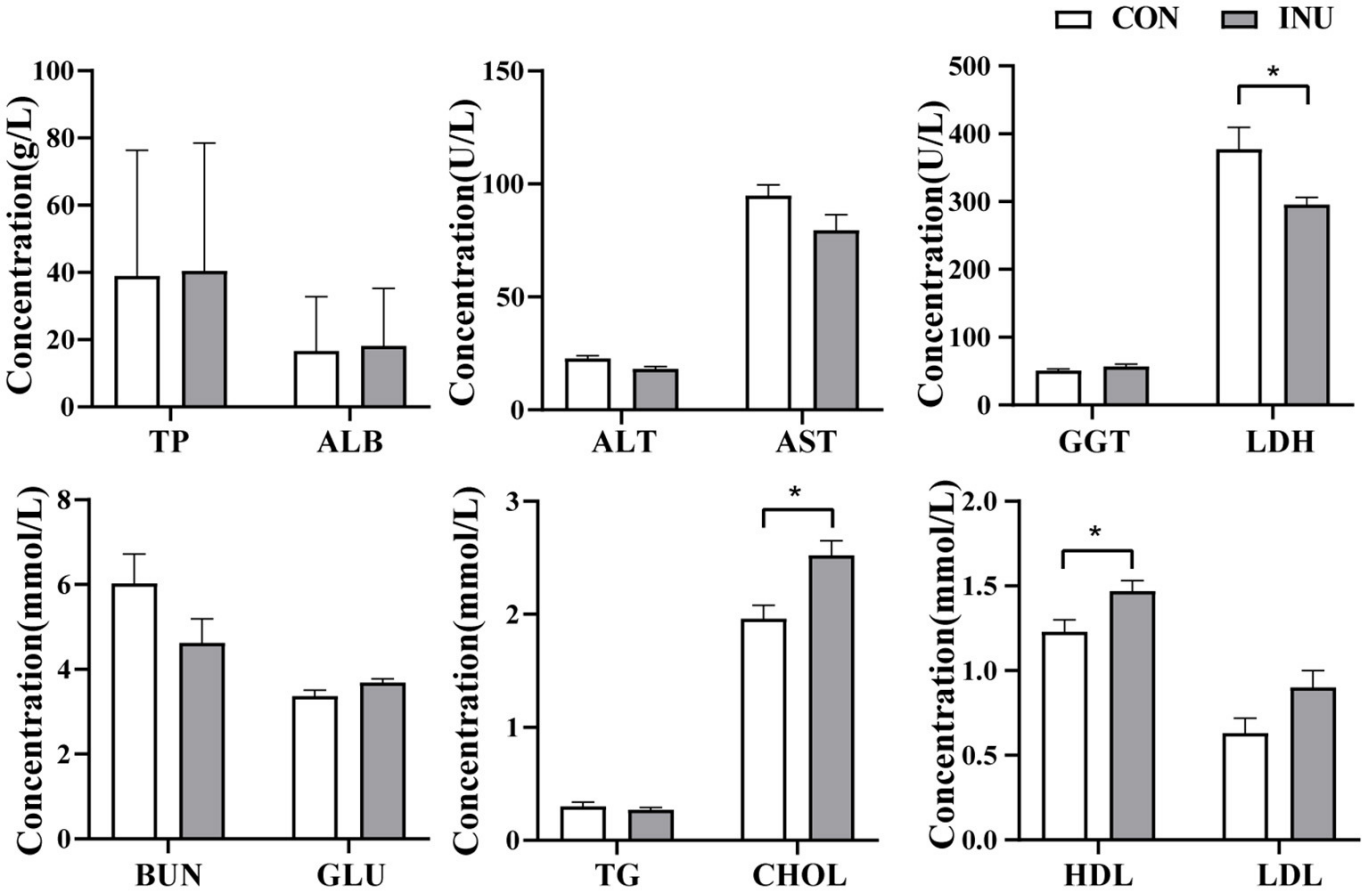
Serum metabolite profile of weaned goats fed maize-grain based (CON) diet or low-carbon high inulin (INU) diet. Values are presented as means±SEM. *Indicates that *p*-value is < 0.05.

Serum metabolites profile was analyzed to determine the influence of the INU diet on liver function in weaned kids. The results showed that the INU diet decreased (*p* < 0.05) the concentration of ALT and LDH, which are closely related to liver damage, and increased (*p* < 0.05) the concentration of CHOL and HDL, which are related to liver metabolism (Figure 2). However, there were no differences (*p* > 0.05) in TP and ALB, related to hepatic synthesis function, between the groups.

### 3.4. Ileal immunity

**Figure 3:**
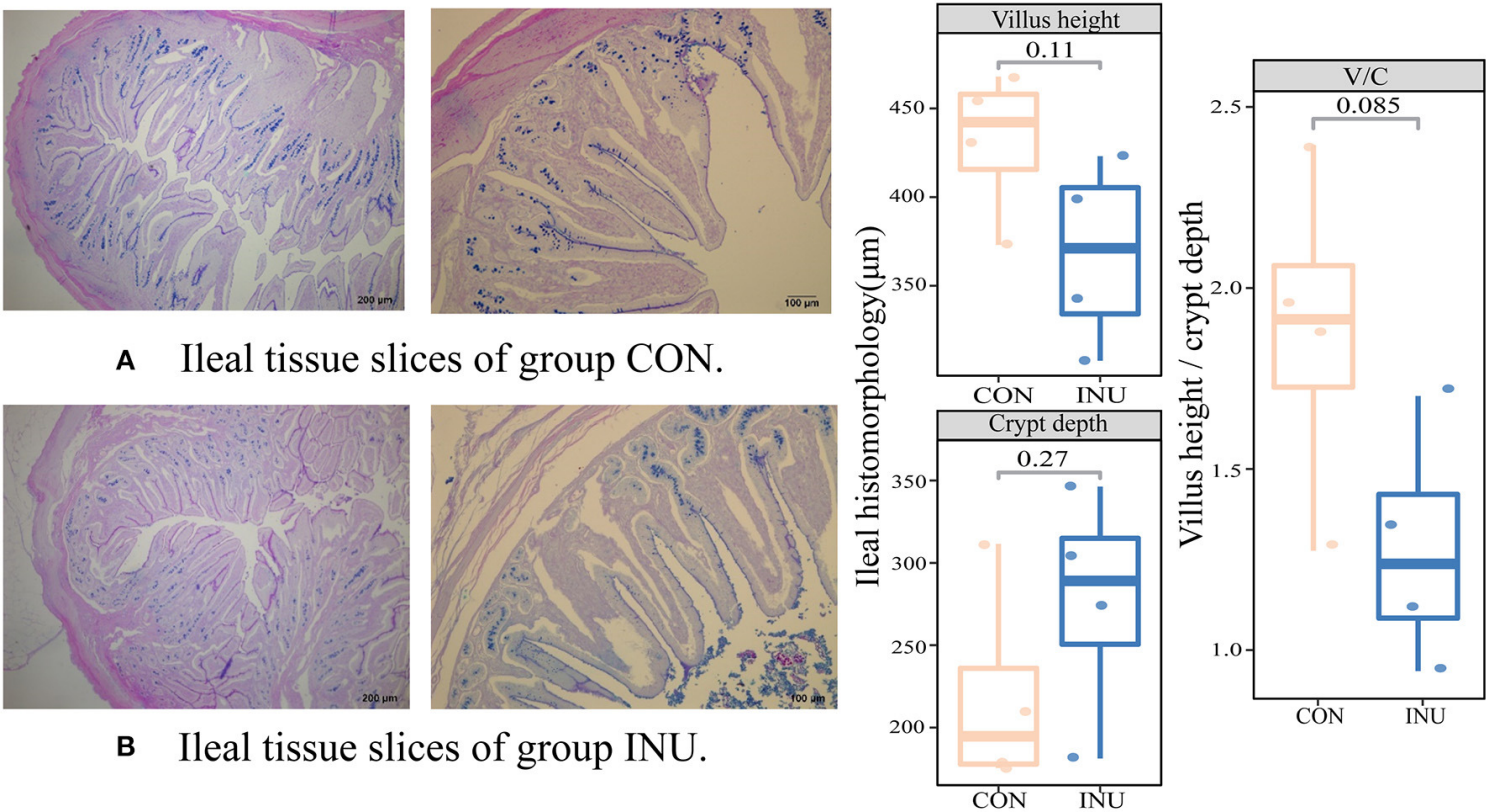
Haematoxylin-eosin staining of and morphological indexes of ileal tissue. **(A)** is the group CON, **(B)** is the group INU. V/C is the ratio of villus height to crypt depth.

**Figure 4:**
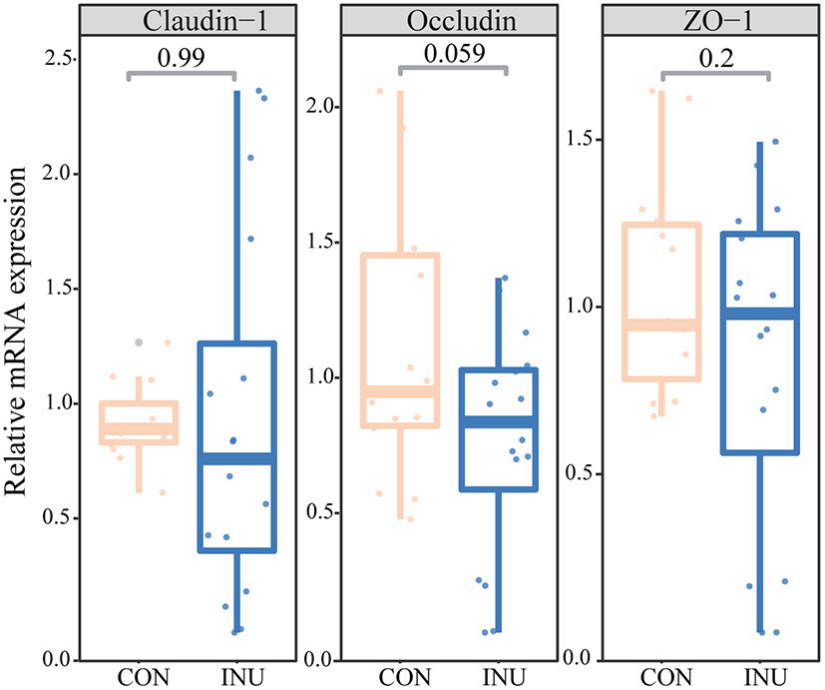
The gene expression of tight junction proteins in weaned goats fed maize-grain based diet (CON) or low-carbon high inulin diet (INU).

Histochemical stained tissue sections, using hematoxylin and eosin (H&E) staining, shows the effect of dietary inulin on ileal tissues (Figure 3). There were no obvious differences in villus (V) height and crypt (C) depth of the ileum in weaned kids fed INU diet compared to the CON group. However, a tendency of reduction in the ratio of V/C (*p* = 0.085) was observed in the INU group. The influence of inulin on intestinal immunity was determined by the underlining changes in expression of genes related to mucosal barrier function, such as *Claudin, Occludin*, and *ZO-1*. There were no differences (*p* > 0.05) in mRNA expression of Claudin, and ZO-1 between the two dietary groups (Figure 4). However, feeding of the INU diet tended (*p* = 0.059) to decrease mRNA expression of Occludin compared with the CON diet.

**Figure 5:**
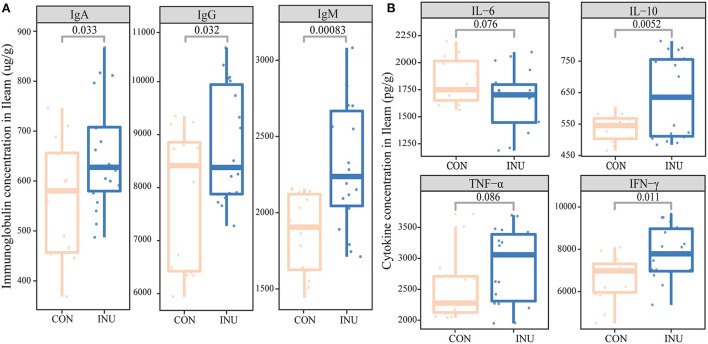
The changes of local immunoglobulins and cytokines in ileum tissue of kids fed maize-grain based diet (CON) or low-carbon high inulin diet (INU). **(A)** is the immunoglobulins (IgA, IgG and IgM), **(B)** is the inflammatory cytokins (TNF-α, INF-γ, IL-6, and IL-10).

We evaluated the changes in immunoglobulin concentrations in response to feeding INU diet in the ileum tissue of weaned kids (Figure 5). The results showed that IgG, IgA, and IgM concentrations were higher (*p* < 0.05) in kids fed INU diet than those fed CON diet. In addition, IgG concentration in the ileum section was higher than IgM and IgA.

In general, cytokines are small proteins secreted by cells and involved in cellular communication and interaction. In the present study, we investigated the effect of dietary inulin on proinflammatory cytokines (TNF-α, INF-γ, and IL-6) and anti-inflammatory cytokines (IL-10) in the ileum tissue of weaned goats. Our findings demonstrated a significant improvement in the levels of the anti-inflammatory cytokines IL-10 (*p* < 0.05) and the proinflammatory cytokines INF-γ (*p* < 0.05) in the ileal tissue of kids fed the INU diet as compared with those fed the CON diet. However, proinflammatory IL-6 levels tended (*p* = 0.076) to decrease in the INU group.

### 3.5. *MTOR* signaling of *longissimus dorsi* muscle

**Figure 6:**
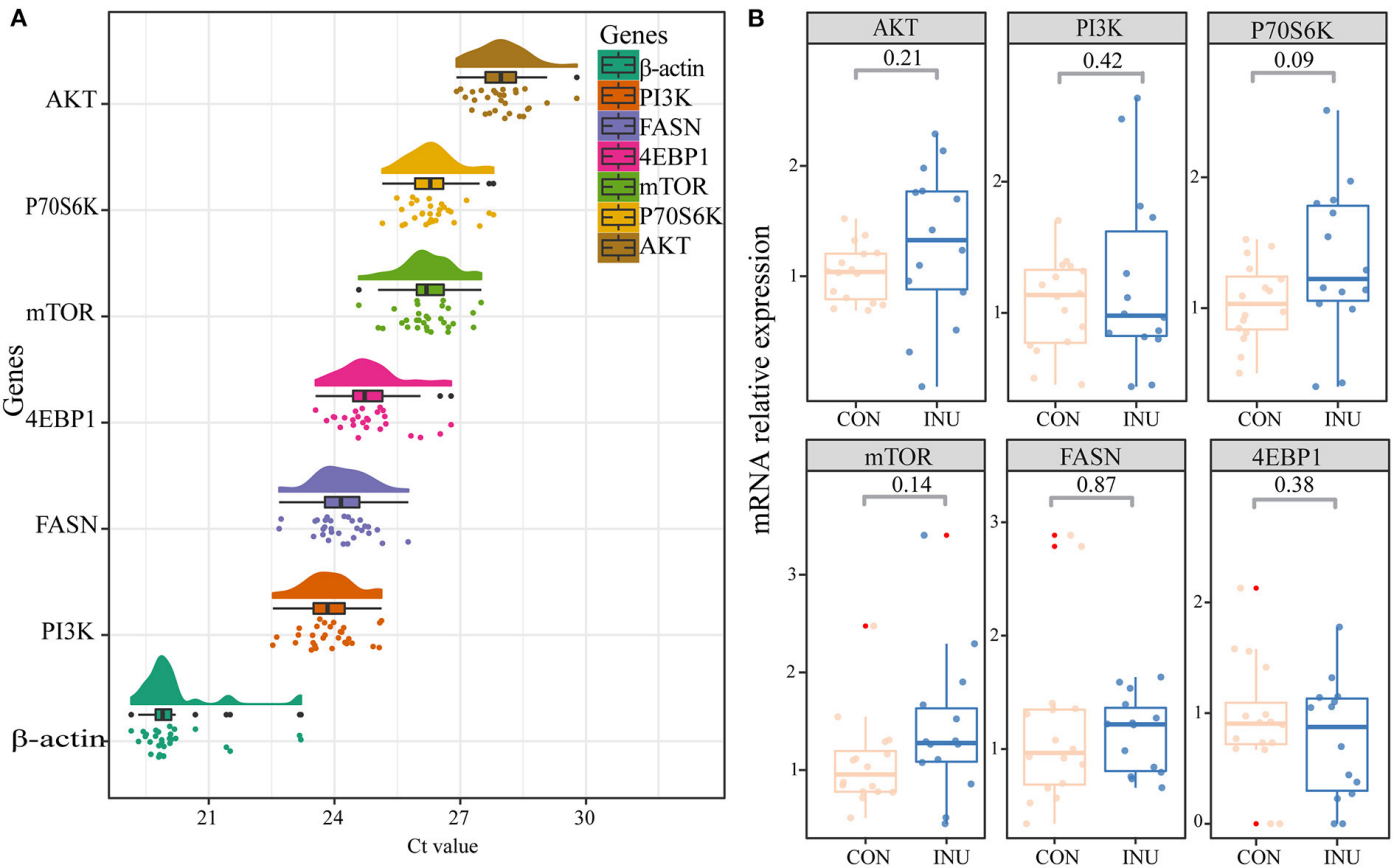
Expression of key genes in mTOR signaling pathway of *longissimus dorsi* muscle of weaned kids fed maize-grain based diet (CON) or low-carbon high inulin diet (INU). **(A)** is the cloud and rain map of gene expression ct value, **(B)** is the mTOR signaling pathway gene expression.

We detected the changes in expression of *PI3K, AKT, mTOR, P70S6K, 4EBP-*1, and *FASN* (*mTOR* pathway) genes *longissimus dorsi* muscle of kids (Figure 6). Our results showed that the relative gene expression of *P70S6K* in the *mTOR* pathway tended (*p* = 0.09) to increase by feeding of the INU diet to kids. However, the expression of *PI3K, AKT, mTOR, 4EBP-*1, and *FASN* genes did not show significant differences (*p* > 0.05) between the two dietary groups.

## 4. Discussion

### 4.1. Methane emission

Methane emissions from ruminants represents a significant loss of digestible energy (5–12%) to the environment, that could be channeled toward animal growth and production (19). Many researchers have found that prebiotics could reduce methane production (19), such as chitosan (20), yeast products (21) and galacto-oligosaccharides (22). It has also been reported that inulin can reduce methane production in ruminants (23). Consistent with the previous findings, the present *in vitro* data showed that inulin-based diets can potentially reduce methane emissions in goats. These findings demonstrated the potential role of inulin in reducing methane emission of ruminant livestock.

### 4.2. Low-carbon high-inulin diet improves growth performance and affects serum metabolite

Dietary supplementation of inulin has been shown to improve growth performance (including ADG, FW, and feed conversion ratio) in white shrimp (Litopenaeus vannamei) (24), broiler chickens (25), and finishing beef cattle (3) at 0.4, 1.0, and 2% (weight/weight), respectively. In the current study, however, we provided the weaned goats with a high percentage of inulin (18.9%) in combination with fat powder (14.4%) compensate for the lower energy and weight loss property of inulin (2). Our results showed that a low-carbon high-inulin diet combined with fat powder improved the ADG in weaned goats while reducing the F/G. The reason may be that the fat content is high, which makes the rate of rumen chyme passing through the digestive tract low, which prolongs the digestion and absorption time of nutrients in the digestive tract, and eventually improves ADG. F/G will ultimately help us reduce feeding costs. The above results demonstrate the potential of inulin and fat powder mixture to improve the growth performance of weaned kids as an unconventional feed resource.

Inulin is a type of resistant starch that exerts systemic effects on animals, including lipid metabolism, liver function, and digestive function. High-density lipoprotein cholesterol (HDL-C) is a kind of good cholesterol, because it can eliminate cholesterol in the blood vessel wall, dredge blood vessels, reduce fat deposits in peripheral tissues and prevent the occurrence of disease. In this study, plasma CHOL and HDL concentrations were significantly higher in weaned kids fed with a low-carbon high-inulin diet than in those fed the CON diet. The addition of inulin can improve the cholesterol transport capacity of the body. The capacity of protein metabolism and the index of liver function damage showed that the fat powder added in the inulin group did not cause inevitable damage to hepatocytes and any metabolic disorders. On the contrary, the low-carbon high-inulin diet significantly reduced the levels of ALT and LDH.

### 4.3. Low-carbon high-inulin diet on intestinal mucosal barrier function

The intestinal epithelial cells establish a permeability barrier between the hostile external environment and the internal milieu, and are responsible for nutrient absorption and waste secretion (26). The VH, CD and V/C ratio can be used as important indicators of intestinal nutrient absorption capacity. We found that feeding inulin (18.9%) to goats had no significant effect on VH, CD, or V/C ratio. A previous study reported that young calves (postnatal d32) intestinal mucosa thickness was significantly increased with feeding of inulin (6 g/d) (27), and addition of 2% inulin to milk replacers significantly reduced VH of the ileum in male calves (28). However, dietary supplementation of high inulin and high fat, did not alter the gene expressions of tight junction proteins in weaning kids in this study. The difference may be that inulin was used in the previous studies as an additive to add a small amount to the diet of animals. In this study, inulin and fat powder were used as the main ingredients, and it is envisaged that fat powder could have also played a role in influencing ileal barrier function.

### 4.4. Low-carbon high-inulin diet improves ileal mucosal immunity

Immunoglobulins play a vital role in mucosal homeostasis that is associated with the regulation of intestinal microbiota and protection against mucosal inflammation (29). Under homeostatic conditions, IgM is the major immunoglobulin isotype in the ileum mucosa of goats. It appears early in response to the initial exposure to an infection and usually reappears, to a lesser extent, after further exposure. It serves as a carrier of a specific protein, or apoptosis inhibitor of macrophages, which is released to facilitate the removal of cell debris or invasive pathogens (30). In the present study, the concentrations of IgA and IgM in the ileum were improved by the inclusion of inulin and fat powder in the diet, demonstrating an improvement in ileum immunity. Recent studies have shown that dietary fiber can directly interact with intestinal immune cell receptors and synergistically affect immune function (31, 32) by stimulating intestinal epithelial cells (33), and dendritic cells (31). However, further studies are needed to elucidate the specific mode of action.

### 4.5. Inflammatory cytokines of inulin

Inflammatory cytokines play vital roles in preventing pathogens invasion and maintaining mucosal homeostasis (34). The IL-10, secreted by various immune cells, is an anti-inflammatory cytokine that serves as an important regulator in preventing proinflammatory responses and maintaining mucosal homeostasis (35). TNF-α is a potent paracrine and endocrine mediator of inflammatory and immune functions (36, 37), causing an increase in intestinal permeability (38). In this study, feeding inulin diet tended to increase the secretion of TNF-α and IFN-γ in the ileum tissue of weaned kids. IFN-γ can regulate the transcription of hundreds of genes by activating intracellular molecular signal pathways (*JAK*/*STAT* pathway) (39), and mediate regeneration and homeostasis of mucosal immunity (40, 41), and various biological processes (41). However, there is no evidence, showing that the influence of inulin on the *JAK/STAT* signaling pathway promotes inflammatory cytokines.

The IL-6 is an interleukin that exerts its action as a proinflammatory cytokine and an anti-inflammatory myokine (42). We found that feeding inulin to weaned kids significantly decreased the concentration of IL-6 and increased concentration of IL-10, which is consistent with previous research (43). However, some studies have also found that dietary inulin supplementation showed no significant effects on the IL-6, IL-8, IL-1β, and TNF-α (3). Furthermore, the inclusion of inulin in pig, mouse, and human diets reduced TNF-α levels (44, 45). Notably, the findings of the present study revealed that TNF-α and INF-γ in the INU group tended to increase with the inclusion of inulin and fat powder in the diet of weaned kids.

A comparison of the findings with those of other studies confirms that inulin can promote the release of some proinflammatory factors (like IL-8, TNF-α) (46). But, inulin has also been reported to reduce inflammation by reducing plasma LPS, IL-6, and TNF-α (43). These results further support the prebiotic function of inulin. There is a large release of proinflammatory factors in the early immune activation in animals, which promotes signal transduction between cells. To maintain the balance in the body, the concentrations of anti-inflammatory and proinflammatory factors maintain a dynamic balance. With production of IL-1, IL-6, and IL-12, and down-regulation of antigen presentation, infection lead to cytokine storm in mice lacking IL-10 (47). Inulin can enhance the immune function of animals, and our findings demonstrate that a diet containing mixture of inulin and fat power can also effectively improve ileal immune function of weaned kids.

### 4.6. Effect of inulin on the *mTOR* pathway

In recent years, dietary fiber (such as inulin) has attracted widespread research interest since it has been found to play a critical role in regulating gut homeostasis and intestinal microflora (48). In our study, the INU group (diet containing 18.9% polyfructan) achieved higher ADG and a lower F/G ratio, indicating a positive effect of inulin on growth regulation in kids. The results were similar to those of previous studies (49). Therefore, we further compared the expression levels of genes (*PI3K, Akt, mTOR, P70S6K, 4EBP*1, *FASN*) involved in fat deposition in the *mTOR* pathway in the *longissimus dorsi* muscle. The *PI3K-AKT-mTOR* participates in normal cell cycle and protein translation (50). FASN, as a rate-limiting enzyme in endogenous fatty acid metabolism, participates in regulating cell growth and differentiation in the *mTOR* pathway (51). In the present study, we found no significant differences in expression of these genes between the two dietary groups, suggesting that changes in weight gain was not caused by high expression of genes associated with fat deposition. Based on increasing expression of fat deposition-related genes in the *mTOR* pathway in our study, subsequent studies on the effects of inulin on animal growth performance should probably pay more attention to genes related to growth hormone, insulin, and other regulatory factors.

## 5. Conclusions

In weaned kids, an inulin-rich diet mainly prevents diseases and improves ileal immune function by increasing the release of immunoglobulins and inflammatory factors rather than regulating tight junction protein expression. Feeding high inulin and fat powder-based energy-rich diet to kids increased ADG by stimulating the expression of *P70S6K*, which modulates *mTOR* signaling and causes fat deposition in muscle. More importantly, the inulin-based diet can reduce the production of methane in the rumen of ruminants. In doing so, we outline the potential of a high-inulin and fat powder diet in modulating intestinal immunity and growth performance of weaned goats under abrupt weaning stress conditions and reducing methane production.

## Data availability statement

The original contributions presented in the study are included in the article/Supplementary material, further inquiries can be directed to the corresponding authors.

## Ethics statement

The animal study was reviewed and approved by the Animal Care Committee, according to the Animal Care and the Use Guidelines of the Institute of Subtropical Agriculture, Chinese Academy of Sciences, Changsha, China.

## Author contributions

CY and XY performed the data analysis and wrote the manuscript. KG performed the animal feeding experiment and some sample analysis. ST, CZ, and ZT ensured the smooth progress of the experiment. SW, NK, and YL revised and reviewed the manuscript. All authors contributed to the article and approved the submitted version.
