# Supplementary figures and images for: A low-carbon high inulin diet improves intestinal mucosal barrier function and immunity against infectious diseases in goats
Source: Front Vet Sci. 2023 Jan 11;9:1098651. doi: 10.3389/fvets.2022.1098651 (PMC9874328; doi:10.3389/fvets.2022.1098651)

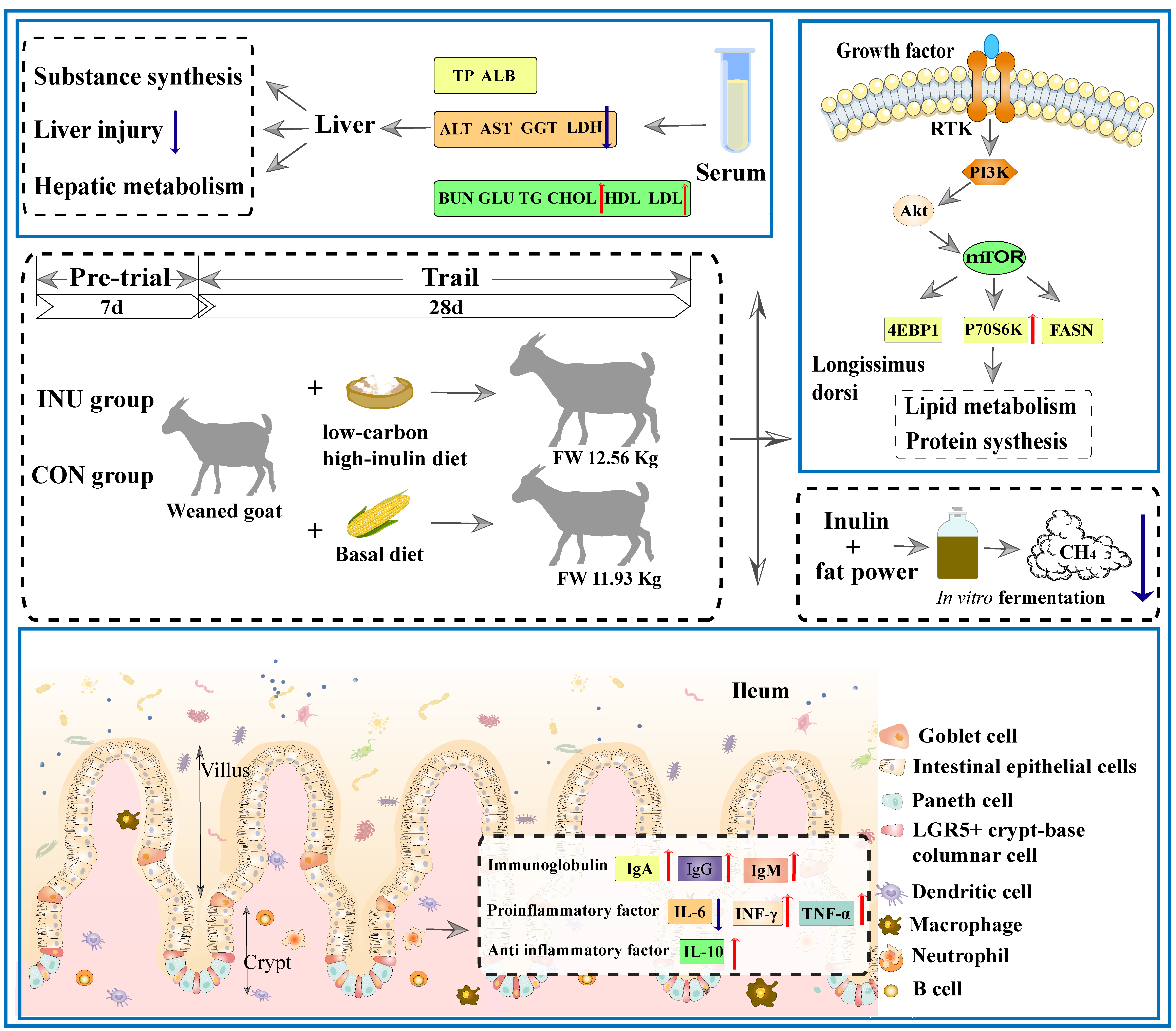

Supplement: Supplementary file 1 [file Image_1.PNG]
